# Extremely Low-Frequency Electromagnetic Fields Promote *In Vitro* Neuronal Differentiation and Neurite Outgrowth of Embryonic Neural Stem Cells via Up-Regulating TRPC1

**DOI:** 10.1371/journal.pone.0150923

**Published:** 2016-03-07

**Authors:** Qinlong Ma, Chunhai Chen, Ping Deng, Gang Zhu, Min Lin, Lei Zhang, Shangcheng Xu, Mindi He, Yonghui Lu, Weixia Duan, Huifeng Pi, Zhengwang Cao, Liping Pei, Min Li, Chuan Liu, Yanwen Zhang, Min Zhong, Zhou Zhou, Zhengping Yu

**Affiliations:** 1 Department of Occupational Health, Faculty of Preventive Medicine, Third Military Medical University, Chongqing, China; 2 Key Laboratory of Electromagnetic Radiation Protection, Ministry of Education of China, Third Military Medical University, Chongqing, China; National Insttitute on Drug Abuse, UNITED STATES

## Abstract

Exposure to extremely low-frequency electromagnetic fields (ELF-EMFs) can enhance hippocampal neurogenesis in adult mice. However, little is focused on the effects of ELF-EMFs on embryonic neurogenesis. Here, we studied the potential effects of ELF-EMFs on embryonic neural stem cells (eNSCs). We exposed eNSCs to ELF-EMF (50 Hz, 1 mT) for 1, 2, and 3 days with 4 hours per day. We found that eNSC proliferation and maintenance were significantly enhanced after ELF-EMF exposure in proliferation medium. ELF-EMF exposure increased the ratio of differentiated neurons and promoted the neurite outgrowth of eNSC-derived neurons without influencing astrocyes differentiation and the cell apoptosis. In addition, the expression of the proneural genes, NeuroD and Ngn1, which are crucial for neuronal differentiation and neurite outgrowth, was increased after ELF-EMF exposure. Moreover, the expression of transient receptor potential canonical 1 (TRPC1) was significantly up-regulated accompanied by increased the peak amplitude of intracellular calcium level induced by ELF-EMF. Furthermore, silencing TRPC1 expression eliminated the up-regulation of the proneural genes and the promotion of neuronal differentiation and neurite outgrowth induced by ELF-EMF. These results suggest that ELF-EMF exposure promotes the neuronal differentiation and neurite outgrowth of eNSCs via up-regulation the expression of TRPC1 and proneural genes (NeuroD and Ngn1). These findings also provide new insights in understanding the effects of ELF-EMF exposure on embryonic brain development.

## Introduction

The potential effects of electromagnetic fields (EMFs) on brain development have raised worldwide public concerns. The frequency of EMFs generated from power lines and household electric appliances is usually 50/60 Hz, which belongs to the extremely low-frequency (ELF) spectrum. It is difficult to avoid such ELF-EMF exposure because they exist wherever electricity is generated, transmitted or used. Moreover, brain development, especially during pregnancy, is particularly sensitive to chemical agents and physical factors [1–3]. However, the results of ELF-EMFs on embryonic brain development are still controversial. Ryan et al found that fetal exposure to ELF-EMF did not induce significant brain anomalies in rats [4]. However, another study reported that fetal ELF-EMF exposure could inhibit the paired-pulse depression and decrease the long-term potentiation in rat brain slices [5]. The 5-HT content was also significantly increased at birth in rat cerebral cortex following ELF-EMF exposure during pregnancy [6]. Therefore, it is still needed to explore the potential effects of ELF-EMF exposure on fetal brain development.

Embryonic neural stem cells (eNSCs), which can develop into three major cell types in the brain [7], play a vital role in embryonic brain development and can imitate the main processes in fetal brain development, such as eNSC proliferation and renewal, neural and glial differentiation, cell migration and neurite outgrowth. eNSCs have therefore become a new model for evaluating the potential effects of chemical agents and physical stimuli on neurodevelopment [3]. Nikolova et al found that ELF-EMF exposure of ES-derived neural progenitor cells transiently up-regulated the mRNA levels of bcl-2 and bax and down-regulated GADD45 mRNA levels. However, the cell physiology, including proliferation, chromosomal stability, and apoptosis, was not altered [8]. In our previous study, we found that intermittent 50 Hz ELF-EMF (5 minutes on and 10 minutes off) exposure did not change eNSCs proliferation or the ratio of neurons and astrocytes derived from eNSCs, but did alter the transcript levels of pro-neuronal genes (one class of bHLH genes) in differentiating eNSCs [9]. Additionally, Grassi and Cuccurazzu et al reported that ELF-EMF exposure (50 Hz, 1 mT, 24 hours per day for about 6 to 10 days *in vitro*; 50 Hz, 1 mT, 3.5 h per day for 12 days *in vivo*) promoted the neurogenesis of postnatal and adult NSCs [10, 11]. It is well known that the exposure condition, such as magnetic intensity, time and period, is one of the most important factors influencing the biological effects of EMF. Here, we wondered whether exposure to ELF-EMF (50 Hz, 1 mT, 4 hours per day, 3 days), similar to Cuccurazzu’s study [11], could alter the development of eNSCs and, if so, to explore the mechanisms underlying this effect.

Many biological effects of ELF-EMF are believed to be mediated by changes in intracellular Ca^2+^ signaling and homeostasis through calcium channels [12–15]. For example, in pituitary cells, ELF-EMF exposure can induce calcium-dependent cell differentiation [16]. Neurite growth promotion through activation of high voltage-activated (HVA) Ca^2+^ channels was observed following ELF-EMF stimulation [17]. It is well known that the main calcium channels are usually classified into voltage operated calcium channels (VOCCs) and non-voltage operated calcium channels (non-VOCCs) [18]. However, eNSCs express few classical VOCCs, which are expressed later during development, indicate that non-VOCCs play an important role in the differentiation of eNSCs [19, 20]. The transient receptor potential canonical (TRPC) family, belonging to non-VOCCs, has been identified in the brain. This family has seven subtypes (TRPC1-7) and plays diverse roles in cell differentiation and neurite extension. In PC12 cells, TRPC1 promoted neurite outgrowth, whereas TRPC5 reduced neurite outgrowth [21]. Calcium regulation by TRPC5 played a key role as a switch between the proliferation and neuronal differentiation of neural progenitor cells [20]. Therefore, we hypothesize that TRPCs participate in embryonic brain development following ELF-EMF exposure.

The present study aimed to determine whether ELF-EMF exposure also affects eNSCs and, if so, to identify the underlying molecular mechanisms. Here, we demonstrate that ELF-EMF exposure induced enhancement of neuronal differentiation and neurite outgrowth of eNSCs is associated with the up-regulation of TRPC1. These results provide a new understanding of the effects of ELF-EMF exposure on embryonic brain development.

## Methods

### Ethics Statement

All experiments were approved by the Ethics Committee of the Third Military Medical University. The animal care and procedures were in accordance with the guidelines of the National Institutes of Health.

### eNSC culture and differentiation

eNSCs were prepared as described in our previous studies [9, 22]. Briefly, telencephalons were isolated from embryonic day 13.5 (E13.5) BALB/c mice. The cells were cultured in proliferation medium composed of a 1:1 (v/v) mixture of Dulbecco’s modified Eagle’s medium (DMEM) and F12 medium (Gibco, USA) with B27 (1×) supplements (Gibco, USA), basic fibroblast growth factor (bFGF) (20 ng/ml; Sigma-Aldrich, USA) and epidermal growth factor (EGF) (20 ng/ml; Sigma-Aldrich, USA). Half of the medium was changed every 3 days. The cultured cells were passaged every 6 days and the second to third passages were used in the experiments. To induce differentiation, eNSCs were cultured in differentiation medium, in which bFGF and EGF were replaced with 1% fetal bovine serum (FBS) (HyClone, USA).

### ELF-EMF exposure

**Fig 1:**
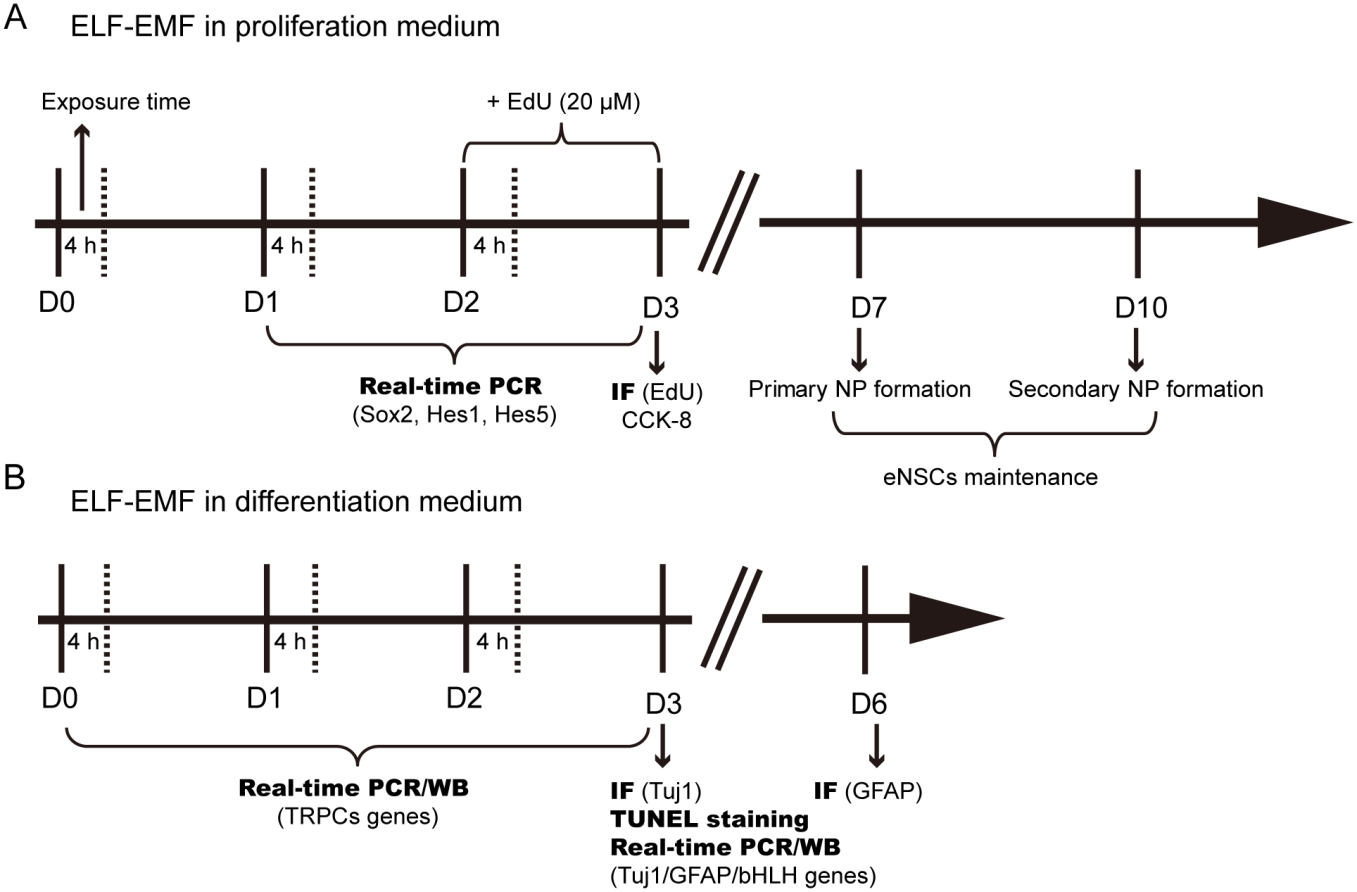
Schematic diagram of the experimental design and exposure time. (**A**) eNSCs cultured in proliferation medium were exposed to ELF-EMF for 3 days (4 hours per day from D0 to D3) and processed for cell proliferation and eNSCs maintenance. (**B**) eNSCs cultured in differentiation medium were exposed to ELF-EMF for 3 days (4 hours per day from D0 to D3) and processed for imumunocytochemical and molecular analyses. ELF-EMF, extremely low-frequency electromagnetic field; IF, imumunofluorescence; WB, western blot.

For all experiments, eNSCs were irradiated in the sXc-ELF exposure system, which was developed and provided by the Foundation for Research and Information Technologies in Society (IT’IS Foundation, Zurich, Switzerland). This system was described in detail previously [23]. Briefly, two identical chambers were placed inside a commercial incubator (HERAcell^®^ 150 i, Thermo Scientific, USA) to ensure stable environmental conditions (37°C, 5% CO2, 95% humidity). The temperature variance between the chambers did not exceed 0.3°C. During exposure, the chambers were randomly assigned by a computer program. One chambers was for the sham control (no radiation), and the other was for the experimental condition (with radiation). As we mentioned in our previous study, the exposure time was set up to 3 days, which is about 2–3 times of population-doubling time in eNSCs [9, 24, 25]. The detailed schedule of ELF-EMF exposure (50 Hz; 1 mT) were applied to evaluate the effects of: (a) in proliferation medium, on the cell viability, EdU incorporation, eNSCs maintenance, the expression of genes (Sox2, Hes1 and Hes5) (4 h/day for 3 days); Fig 1A); (b) in differentiation medium, on the expression of related genes and proteins (4 h/day for 1 day, 2 days or 3 days); Fig 1B).

### Cell viability assay

Cell viability was determined by a Colorimetric Cell-counting Kit (CCK-8; Dojindo, Japan) assay according to the manufacturer’s instructions. Briefly, eNSCs (1.0×10^4^ cells in 100 μl proliferation medium) were cultured in a 96-well plate and immediately exposed to ELF-EMF for 3 days. Then, the CCK-8 solution (10 μl) was added into the medium (100 μl) and incubated at 37°C for 3 hours. The OD value was measured at 450 nm with a microplate reader (SPECTRA-FLUOR, TECAN, Sunrise, Austria).

### EdU incorporation

EdU incorporation was used to detect the cell proliferation as previous described [9]. Briefly, eNSCs (1.0×10^5^ cells/ml) were exposed to ELF-EMF for 3 days. EdU (20 μM) was added to the culture medium at the last 24 h. Then, the cells were plated onto poly-L-lysine-coated coverslips for attachment and fixed with 4% paraformaldehyde. After that, cells were rinsed with PBS and stained with the staining mix for 30 min at room temperature. After staining, the cells were washed 3 times with PBS. Cell nuclei were counterstained with Hoechst 33342 (5 μg/ml; Sigma-Aldrich, USA). The number of EdU-positive and total cells was counted using fluorescence microscopy (Leica, Germany) in four non-overlapping fields per coverslip. The results were expressed as percentages of the total cell numbers.

### Neurosphere Assays

The neurosphere assays were carried out as previously described [22]. The neurosphere assays usually include the neurosphere forming assay and the self-renewal assay. For the neurosphere forming assay, the single eNSCs (1000 cells in 200 μl proliferation medium) were cultured in a 96-well plate and immediately exposed to ELF-EMF for 3 days. After another 4 days of culture, the number of the total number of neurospheres in each well was counted.

To evaluate the self-renewal capacity of eNSCs, the dissociated eNSCs were allowed to form neurospheres with ELF-EMF exposure for 3 days. Then the new generated neurospheres were collected and dissociated into single cells. The cell suspension (1000 cells in 200 μl proliferation medium) was transferred to each well of a 96-well plate without ELF-EMF exposure and the number of neurospheres in each well was counted after 7 days.

### Immunocytochemistry and cell counts

eNSCs (1.0×10^5^ cells in 1 ml) were plated onto poly-L-lysine-coated glass coverslips and were exposed to ELF-EMF for 3 days in differentiation medium. Then the cells were immediately fixed with 4°C 4% paraformaldehyde for 20 min (for neuron staining) or fixed after another 3 days of culture (for astrocytes staining). Immunocytochemistry was carried out as previously described [26]. The primary antibodies of mouse anti-mouse Tuj1 (1:100, R&D, USA) and rabbit anti-mouse GFAP (1:200, Beijing Zhongshan, Beijing, China) were used to stain the neurons and astrocytes, respectively. The other applied primary antibodies were as follow: rabbit anti-mouse TRPC1 (1:100, Abcam, USA), rabbit anti-mouse NeuroD (1:100, Santa Cruz, USA), goat anti-mouse Ngn1 (1:50, Santa Cruz, USA). Alexa Fluor 488-labelled donkey anti-mouse, Alexa Fluor 555-labelled donkey anti-rabbit and Alexa Fluor 647-labelled donkey anti-goat secondary antibodies (1:200, Invitrogen, USA) were used for visualization. Cell nuclei were stained with Hoechst33342 (5 μg/ml). Tuj1^+^ and GFAP^+^ cells were counted in four different fields of each coverslip using a 63×objective under a Leica TCS SP5 confocal fluorescence microscope. More than 1000 cells on 12 coverslips from five independent experiments were counted.

### Neurite outgrowth analysis

The neurite outgrowth was analyzed as previously described [27]. Briefly, eNSCs (1000 cells in 1 ml medium) were plated onto 15 mm glass coverslips and exposed to ELF-EMF for 3 days in differentiation medium. Then, the cells were fixed with 4% paraformaldehyde for immunofluorescence staining for Tuj1. Images were acquired using a Leica microscope by an experimenter who was blinded to the exposure condition. Three parameters of the neurite outgrowth of Tuj1^+^ neurons were analyzed using the Image J software: the total length of the neurites per cell, the number of primary neurites per cell and the number of branch points per cell. Neurite length was assessed by measuring the total length from one cell body to the end of all neuritis. The final length was the sum of all neurites that were measured from this one cell body. The neurons in these fluorescence images that overlapped with neighboring neurons were excluded from this analysis. Images of at least 30 neurons per condition were captured for each experiment, and five independent experiments were performed.

### TUNEL staining

The differentiating eNSCs were plated onto poly-L-lysine-coated coverslips and exposed to ELF-EMF for 3 days. The cells were then fixed with 4% paraformaldehyde for 20 min. Cell apoptosis was detected by TUNEL staining using an in situ cell death detection POD kit (Roche, USA) according to the manufacturer’s instructions. TUNEL^+^ cells were counted in four non-overlapping fields per coverslip using a fluorescence microscope. The results were expressed as percentages of the total cell numbers.

### Real-time PCR

Total RNA was isolated from differentiating eNSCs after ELF-EMF exposure using TRIzol reagent (Takara, Japan). The cDNAs were obtained by reverse transcription PCR. Real-time PCR was performed on a Bio-Rad IQ5 Detection System using SYBR Master Mix (Takara, Japan). The specific gene primers are listed in Table 1. The fold-change in gene expression was calculated using the 2^-ΔΔCT^ method as previously described and was normalized to endogenous GAPDH [28]. The relative gene expression levels were calculated in reference to the sham groups.

**Table 1:** Primers used in real-time PCR analyses.

Gene	Forward primer	Reverse primer
Bax	5′-gca cgt cca cga tca gtc ac-3′	5′-cct gga tga aac cct gta gca-3′
Bcl-2	5′-tgg gat gcc ttt gtg gaa ct-3′	5′-gag aca gcc agg aga aat caa ac-3′
Tuj1	5′-cgc cat gtt cag acg caa g-3′	5′-ctc gga cac cag gtc gtt ca-3′
NeuroD	5′-aca aca gga agt gga aac atg acc-3′	5′-cac tca tct gtc cag ctt ggg-3′
Mash1	5′-act tga act cta tgg cgg gtt-3′	5′-cca gtt ggt aaa gtc cag cag-3′
Math1	5′-gag tgg gct gag gta aaa gag t-3′	5′-ggt cgg tgc tat cca gga g-3′
Math3	5′-ctc tta tgg aat gct cgg aac c-3′	5′-aat ctt tca agg cga gct tta gtc-3′
Ngn1	5′-cca gcg aca ctg agt cct g-3′	5′-cgg gcc ata ggt gaa gtc tt-3′
Ngn2	5′-gtc atc ctc caa ctc cac gtc-3′	5′-agg cgc ata acg atg ctt ctc-3′
Hes1	5′-gaa gag gcg aag ggc aag aa-3′	5′-gag gtg ctt cac agt cat ttc ca-3′
Hes5	5′-gac cgc atc aac agc agc at-3′	5′-ggc gaa ggc ttt gct gtg t-3′
Hes6	5′-cct ggt gga gaa gaa gcg ac-3′	5′-ttg gcc tgc acc tcg gta-3′
Sox2	5′-aac cga tgc acc gct acg a-3′	5′-tgc tgc gag tag gac atg ctg-3′
TRPC1	5′-gga tgt gtc ttt gcc caa gc-3′	5′- ctg gac tgg cca gac atc ta-3′
TRPC3	5′-gta ttc aac gcc tcg gac aga t-3′	5′-cag acc aca tca tcc caa gaa c-3′
TRPC4	5′-ggc ttg acg gag gag aat gtt a-3′	5′-gcg ttg gct gac tgt att gta gag-3′
TRPC5	5′-gca cct tac cac ctc ctt tca ac-3′	5′-ctt cgt ctt cca tca ggg tct ct-3′
TRPC6	5′-gag aac act tcc tgt ccc ctt c-3′	5′-ctt gct ttt gac cct gga tga g-3′
TRPC7	5′-ccg act ggc caa cat tga ga-3′	5′-cca cct ctt ctg tgt ctc gg-3′
GAPDH	5′-ata cgg cta cag caa cag gg-3′	5′-gcc tct ctt gct cag tgt cc-3′

### Western blot analysis

Western blot analysis was performed as described by M He, et al [29]. After ELF-EMF exposure, protein samples were harvested and lysed in RIPA buffer (Thermo Scientific, USA) containing a cocktail of protease inhibitors (Roche, USA). The protein concentration was determined using a BCA protein assay kit (Takara, Japan). Equal amounts of protein were load for SDS-PAGE. After electrophoresis, the proteins were transferred onto a polyvinylidene fluoride membrane (Bio-Rad, USA). Then, the membranes were blocked and incubated with various primary antibodies at 4°C overnight. Mouse anti-mouse TRPC1 (1:1000, Santa Cruz, USA), rabbit anti-mouse NeuroD (1:500, Santa Cruz, USA), goat anti-mouse Ngn1 (1:500, Santa Cruz, USA), and mouse anti-mouse GAPDH (1:5000, Abcam, USA) were used as primary antibodies. The next day, the membranes were washed in Tris-buffered saline-Tween 20 to remove unbound primary antibodies, incubated with the appropriate horseradish peroxidase-conjugated secondary antibodies (1:1000, Beyotime, China), and finally examined using an electrochemiluminescence system (Thermo Fisher Scientific, USA). The bands were imaged and analyzed using a ChemiDoc XRS + System with Image Lab Software (Bio-Rad, USA).

### Measurement of [Ca^2+^]_i_

The [Ca2^+^]_i_ was detected according to the previous study in our lab [30]. Briefly, the cells were seeded into poly-L-lysine-coated 96-well plates and exposed to ELF-EMF with and without the TRPC1-siRNA treatment in differentiation medium. After ELF-EMF exposure for 3 days, the cells were loaded with HEPES-buffered salt solution (HBSS) containing Fura 2-AM (5 μM, Dojindo, Japan) and 0.01% pluronic acid-127 (Sigma-Aldrich, USA) for 45 min at 37°C and then incubated in fresh HBSS for another 30 min to allow deesterification of the Fura-2 AM. Next, the cells were exposed to the 1 mT ELF-EMF for 30 min, and then the intracellular calcium was immediately measured using an Infinite M200 Microplate Reader (TECAN, Austria). The excitation wavelength was set at 340 nm and 380 nm, respectively. The fluorescence emission was monitored at 510 nm. The ratio of the fluorescence intensity at 340 nm to that at 380 nm was calculated to represent the peak amplitude of [Ca^2+^]_i_.

### siRNA transfection

eNSCs were plated onto poly-L-lysine-coated glass coverslips or Petri-dishes (35 mm in diameter) in the serum-free medium. The cells were then transfected with TRPC1-siRNA (Santa Cruz, USA) or control-siRNA (Santa Cruz, USA) using optimum-minimum essential medium with Lipofectamine 2000 (Invitrogen, USA) for 3 days. The knockdown efficiency of TRPC1-siRNA was determined by real-time PCR and Western blot. Next, the cells were exposed to ELF-EMF for 3 days in differentiation medium. After that, the mRNA expression of NeuroD and Ngn1 was measured using real-time PCR. The neuronal ratio and the neurite outgrowth of eNSC-derived neurons were measured as previously described.

### Statistics

All data were expressed as the mean ± standard error of the mean (SEM) from five independent duplicate experiments. The data were analyzed by non-parametric tests with Mann-Whitney and Kruskal-Wallis tests. The level of significance was set at 0.05.

## Results

### ELF-EMF exposure enhances cell proliferation and maintenance

**Fig 2:**
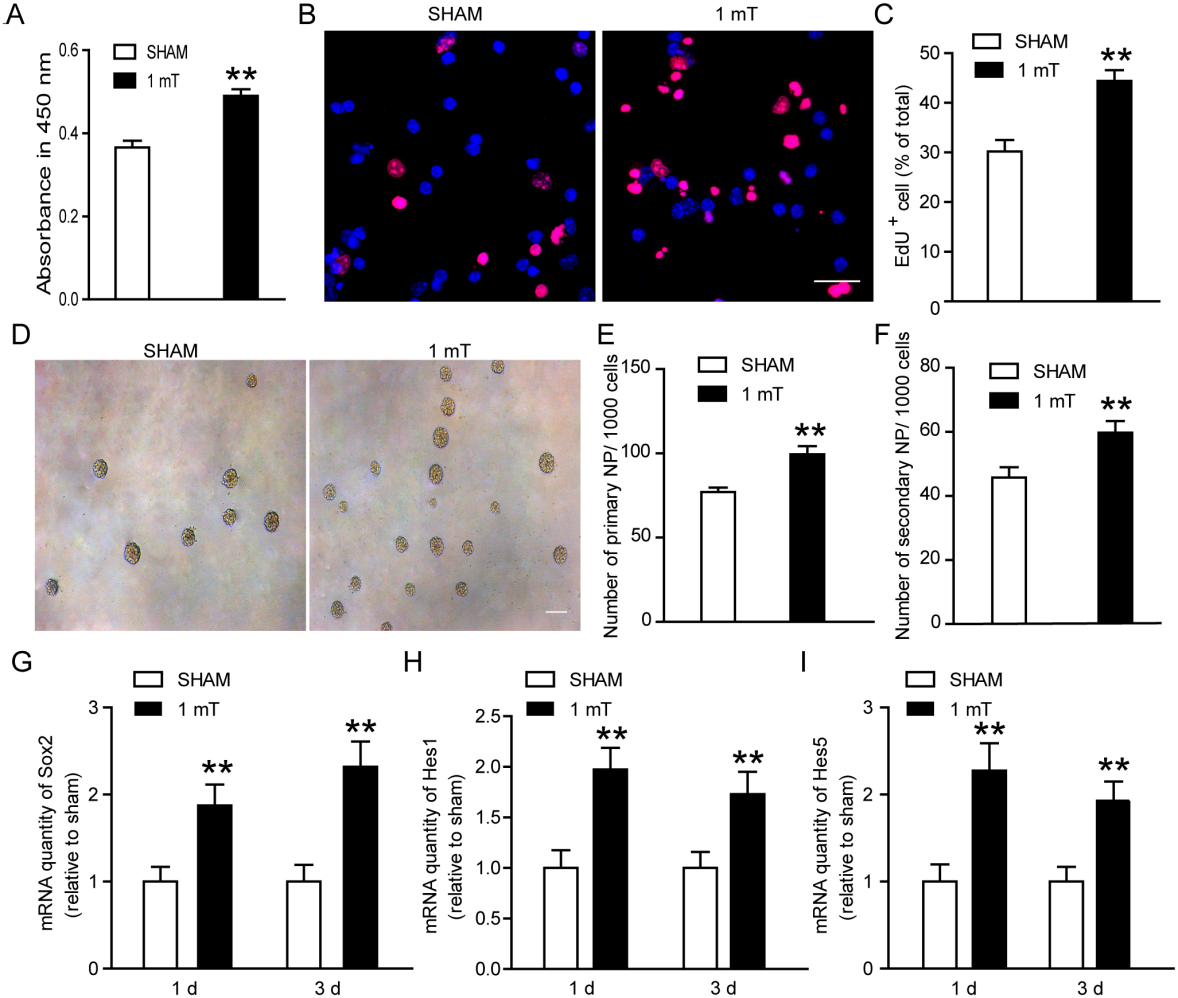
Effects of ELF-EMF exposure on cell proliferation and maintenance of eNSCs. eNSCs were cultured in proliferation medium and exposed to ELF-EMF for 3 days. (**A**) The cell viability was examined by CCK-8 assay. ** *p* < 0.01 vs. sham groups. (**B**) Representative images of EdU staining for cell proliferation. Scale bar: 25 μm. (**C**) The graph showed the statistical results of the percentage of EdU^+^ cells. ** *p* < 0.01 vs. sham groups. (**D**) Representative images of neurospheres for eNSCs maintenance. Scale bar: 100 μm. (**E-F**) The graphs showed the statistical results of numbers of primary neurospheres and secondary neurospheres, respectively. ** *p* < 0.01 vs. sham groups. (**G-I**) The mRNA expression of Sox2, Hes1 and Hes5 after ELF-EMF exposure for 1 day and 3 days. ** *p* < 0.01 vs. sham groups. For all experiments, the data are from five independent experiments and are presented as the mean ± SEM.

Firstly, we analyzed the effects of ELF-EMF exposure on the proliferation of eNSCs. Briefly, eNSCs were exposed to ELF-EMF (1 mT, 4 hours per day) for 3 days, and the cell proliferation were evaluated by CCK-8 assay and EdU incorporation. We found that the cell viability was significantly increased after ELF-EMF exposure (Fig 2A). Meanwhile, the percentage of EdU^+^ cells was also increased in the exposure group as compared to the sham group (Fig 2B and 2C). These results suggest that ELF-EMF (1 mT, 4 hours per day for 3 days) exposure could enhance eNSCs proliferation.

Then, we explored the effect of ELF-EMF on eNSC maintenance via the neurosphere assays. As described in the methods, eNSCs were cultured in suspension at a low density (1000 cells in 200 μl) and most of the newly formed neurospheres were thought to originate from single cells. At first, we detected the effects of ELF-EMF exposure on the neurosphere forming. The single eNSCs were cultured in a 96-well plate and immediately exposed to ELF-EMF for 3 days. After another 4 days of culture, the number of generated neurospheres was obvious increased in the ELF-EMF group (Fig 2E). To explore the effects of ELF-EMF exposure on the self-renewal capacity of eNSCs, we exposed the dissociated eNSCs to ELF-EMF for 3 days. Then the new generated neurospheres were dissociated into single cells and plated in the culture medium to allow the formation of neurospheres without ELF-EMF exposure. After 7 days of culture, we found a markedly increase in the number of generated neurospheres (Fig 2F). Additionally, the results of real-time PCR revealed significant up-regulation in proliferation-related genes (Sox2, Hes1 and Hes5) (Fig 2G–2I) after ELF-EMF exposure for 1 day and 3 days, respectively. Collectively, these results suggest that ELF-EMF exposure could enhance eNSCs proliferation and maintenance.

### ELF-EMF exposure increases the ratio of eNSCs-differentiated neurons

**Fig 3:**
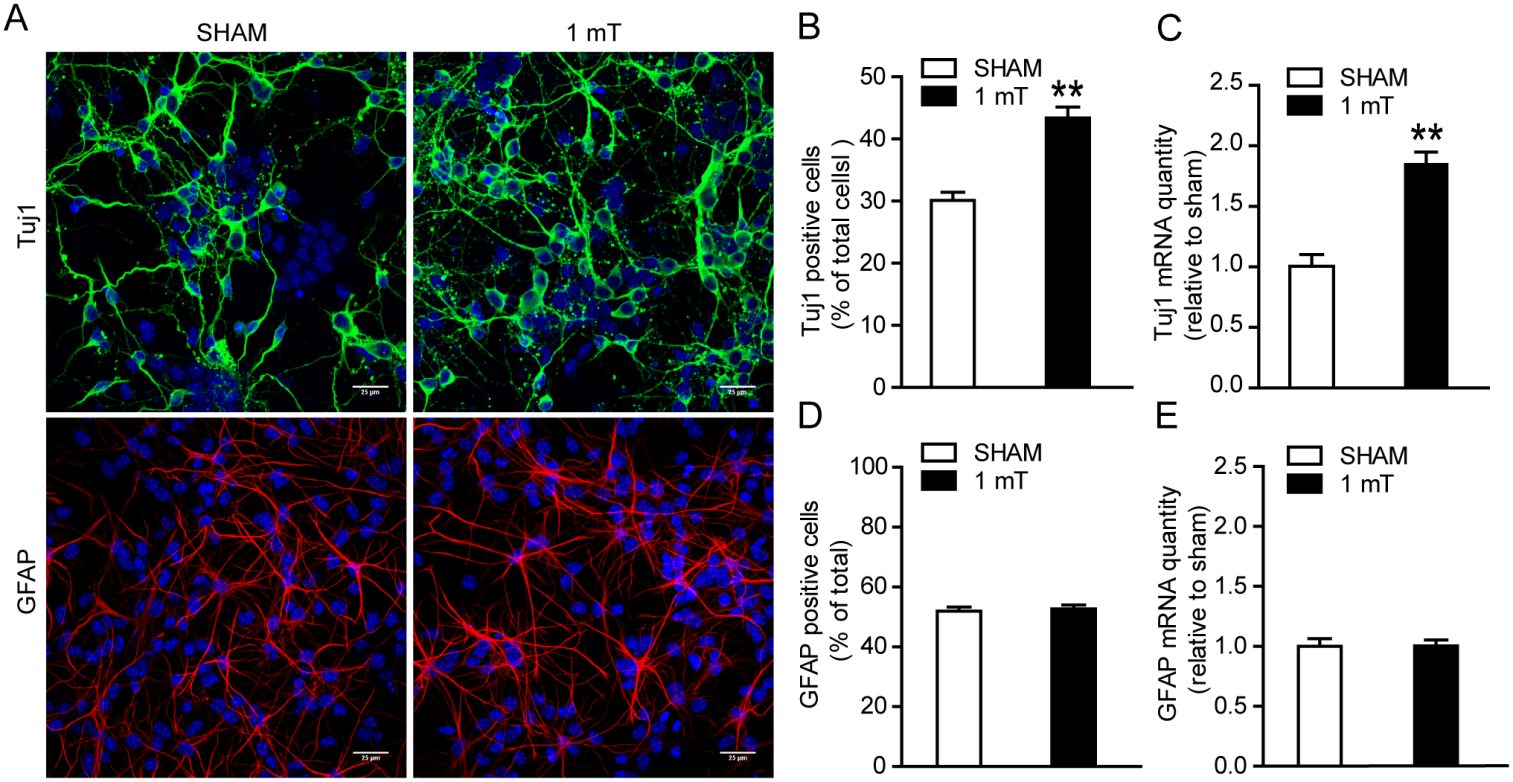
Effects of ELF-EMF exposure on eNSCs differentiation. eNSCs were cultured in differentiation medium and exposed to ELF-EMF for 3 days. The cells were then fixed for Tuj1 (a neuronal marker) staining or cultured for another 3 days for GFAP (an astrocyte marker) staining. The mRNA expression of Tuj1 and GFAP was measured after ELF-EMF exposure for 3 days by real-time PCR. (**A**) Representative images of Tuj1^+^ and GFAP^+^ cells derived from eNSCs. Scale bar: 25 μm. (**B, D**) The graphs showed the statistical results of the percentage of Tuj1^+^ and GFAP^+^ cells in each condition. ** *p* < 0.01 vs. sham groups. (**C, E**) The mRNA expression of Tuj1 and GFAP. ** *p* < 0.01 vs. sham groups. For all experiments, the data are from five independent experiments and are presented as the mean ± SEM.

Since cell differentiation is an important process in eNSCs behavior, the ratio of eNSCs-differentiated neurons and astrocytes was also detected. eNSCs were exposed to ELF-EMF for 3 days in differentiation medium. Then, eNSCs-differentiated neurons were stained with Tuj1 antibody, which is expressed in new generated neurons and can exist for a relative long time [31]. We found a markedly increase in the ratio of eNSCs-differentiated neurons (Fig 3A and 3B). For the astrocytes staining, the cells were cultured for another 3 days after ELF-EMF exposure to allow the full differentiation of astrocytes. No alteration in the percentage of differentiated GFAP^+^ cells was observed (Fig 3A and 3D). Additionally, the mRNA expression of Tuj1 and GFAP were also detected by real-time PCR after ELF-EMF exposure for 3 days. We found that the Tuj1 mRNA expression was significantly up-regulated in the ELF-EMF exposure group, while the GFAP mRNA expression was not changed (Fig 3C and 3E). These results suggest that ELF-EMF exposure could alter eNSCs differentiation and the process of neuronal differentiation of eNSCs is more sensitive than astrocyte differentiation.

### ELF-EMF exposure promotes the neuritis outgrowth of eNSCs-differentiated neurons

**Fig 4:**
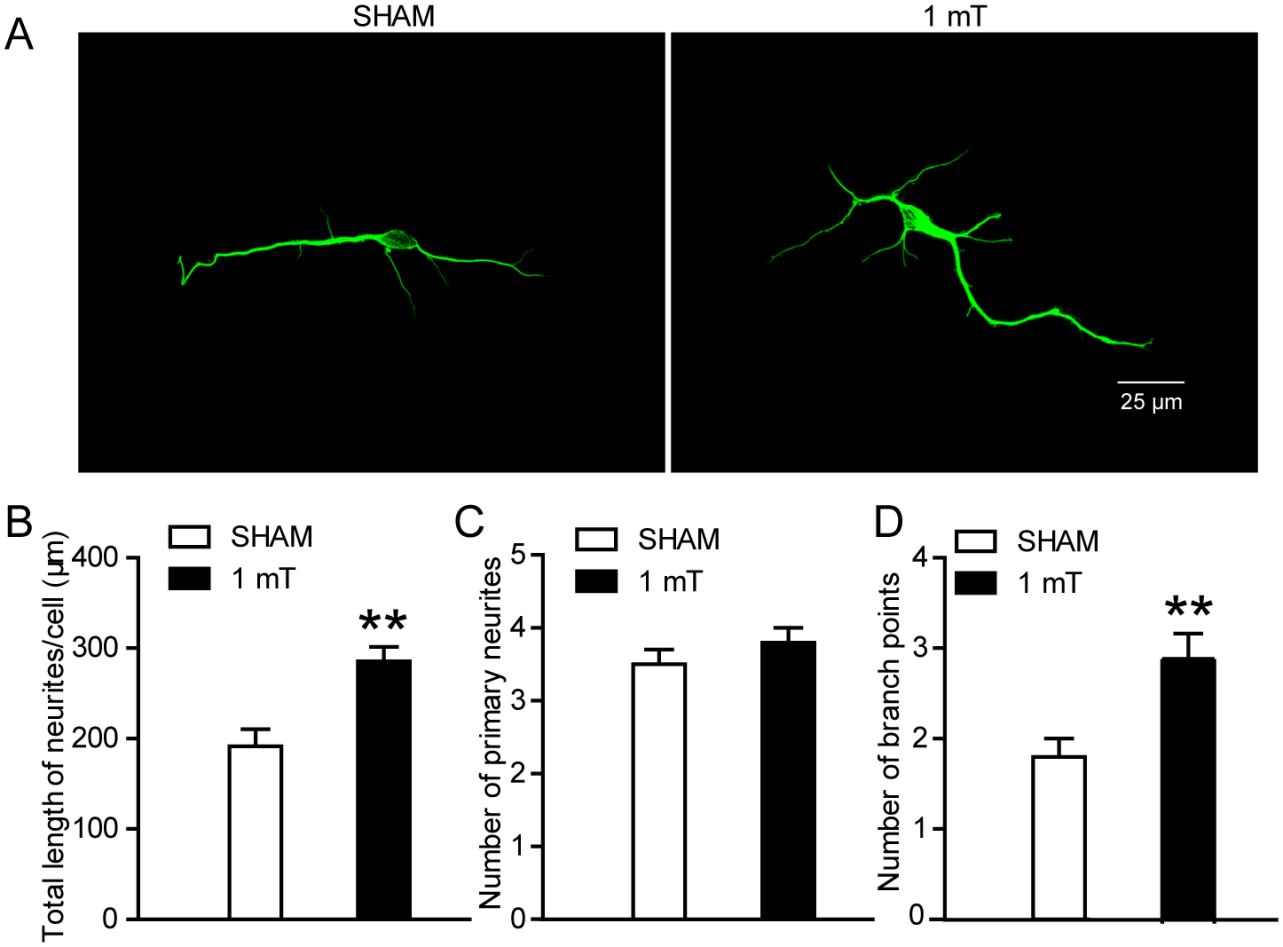
Effects of ELF-EMF exposure on neurite outgrowth of eNSCs-derived neurons. eNSCs were cultured in differentiation medium and exposed to ELF-EMF for 3 days. The neurons derived from eNSCs were stained with Tuj1. (**A**) Representative images of Tuj1 staining for the neurite outgrowth of eNSC-derived neurons. Left pane: The representative image from the sham group. Right pane: The representative image from the ELF-EMF group. Scale bar: 25 μm. (**B-D**) The graphs showed characteristics of the neurites, including the total length of the neurites per cell (**B**), the number of primary neurites per cell (**C**) and the number of branch points per cell (**D**), respectively. ** *p* < 0.01 vs. sham groups. For all experiments, the data are from five independent experiments and are presented as the mean ± SEM.

To further explore the effects of ELF-EMF exposure on the neuronal differentiation of eNSCs, the neuritis outgrowth of eNSCs-differentiated neurons was analyzed. We used three parameters to analyze the outgrowth of neurites: the total length of neurites per cell, the number of primary neurites per cell and the number of branch points per cell [32, 33]. We found that both the total length of neuritis per cell and the number of branch points per cell were markedly increased after ELF-EMF exposure for 3 days, but the number of primary neurites per cell was not altered (Fig 4A–4D). These findings suggest that ELF-EMF exposure could enhance neurite outgrowth of eNSCs.

### ELF-EMF exposure does not induce apoptosis of differentiating eNSCs

**Fig 5:**
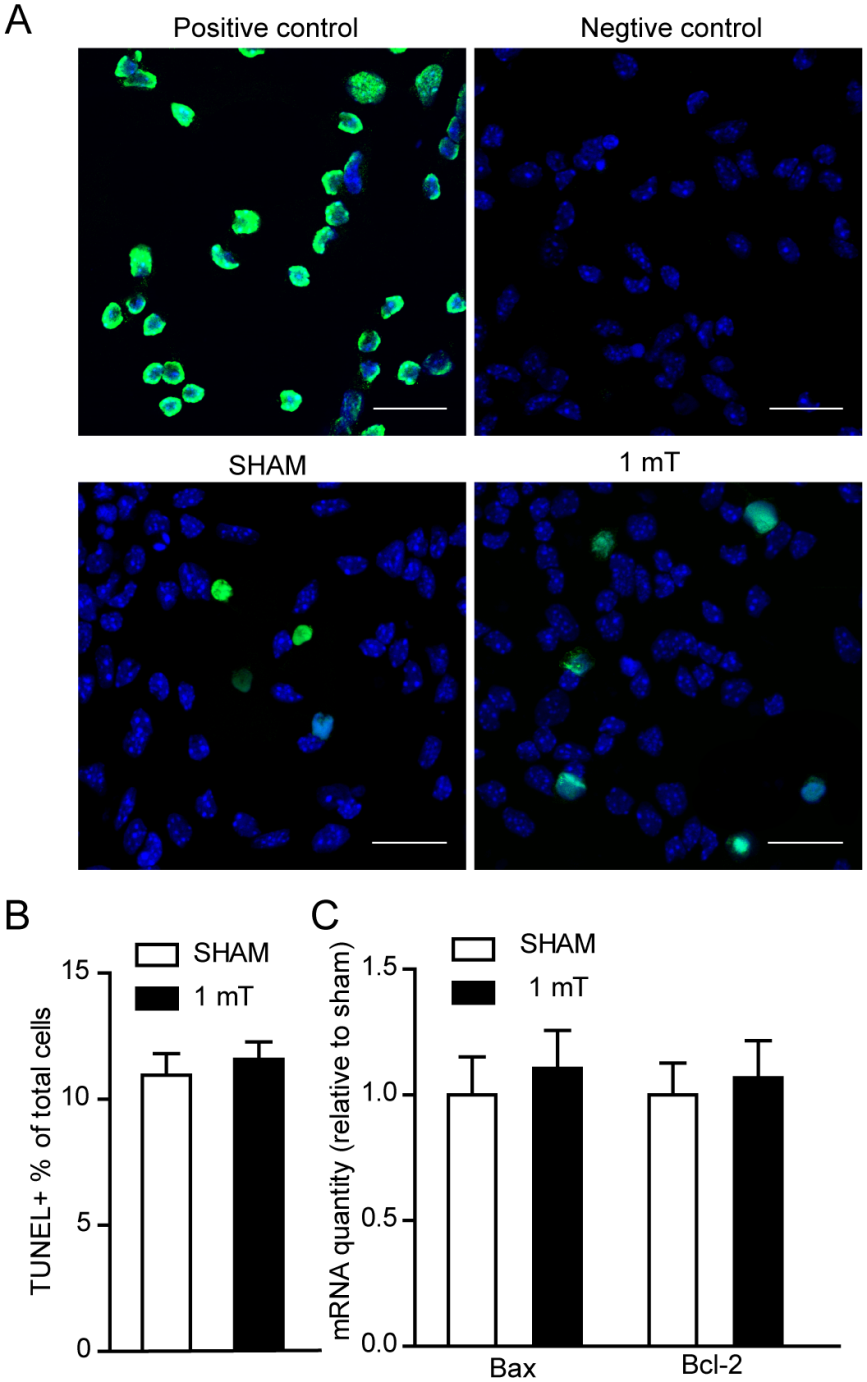
Effects of ELF-EMF exposure on apoptosis of differentiating eNSCs. eNSCs were cultured in differentiation medium and exposed to ELF-EMF for 3 days. (**A**) Representative images of TUNEL staining for cell apoptosis. In the positive control, DNase I was used to damage DNA before the addition of the TdT reaction buffer and all the cells are TUNEL^+^ (green). In the negative control, the TdT reaction buffer was not added, and all the cells are TUNEL^-^. Scale bar: 25 μm. (**B**) Statistical results of the percentage of TUNEL^+^ cells. (**C**) No alteration was observed in the Bax and Bcl-2 mRNA expression, as measured using real-time PCR. For all experiments, the data are from five independent experiments and are presented as the mean ± SEM.

To detect whether the cell apoptosis are affected during eNSCs differentiation, the cell apoptosis was detected using terminal deoxynucleotidyl transferase-mediated biotinylated UTP nick end labeling (TUNEL) kit. Differentiating eNSCs were exposed to ELF-EMF for 3 days. No changes were observed in the percentage of TUNEL^+^ cells between the sham group and the exposure group (Fig 5A and 5B). To further confirm this result, the mRNA expression of the pro-apoptotic gene Bax and the anti-apoptotic gene Bcl-2 was also measured using real-time PCR. No changes were found in either gene (Fig 5C). These results suggest that exposure to ELF-EMF exerts no obvious cell apoptosis on differentiating eNSCs.

### ELF-EMF exposure up-regulates proneural genes

**Fig 6:**
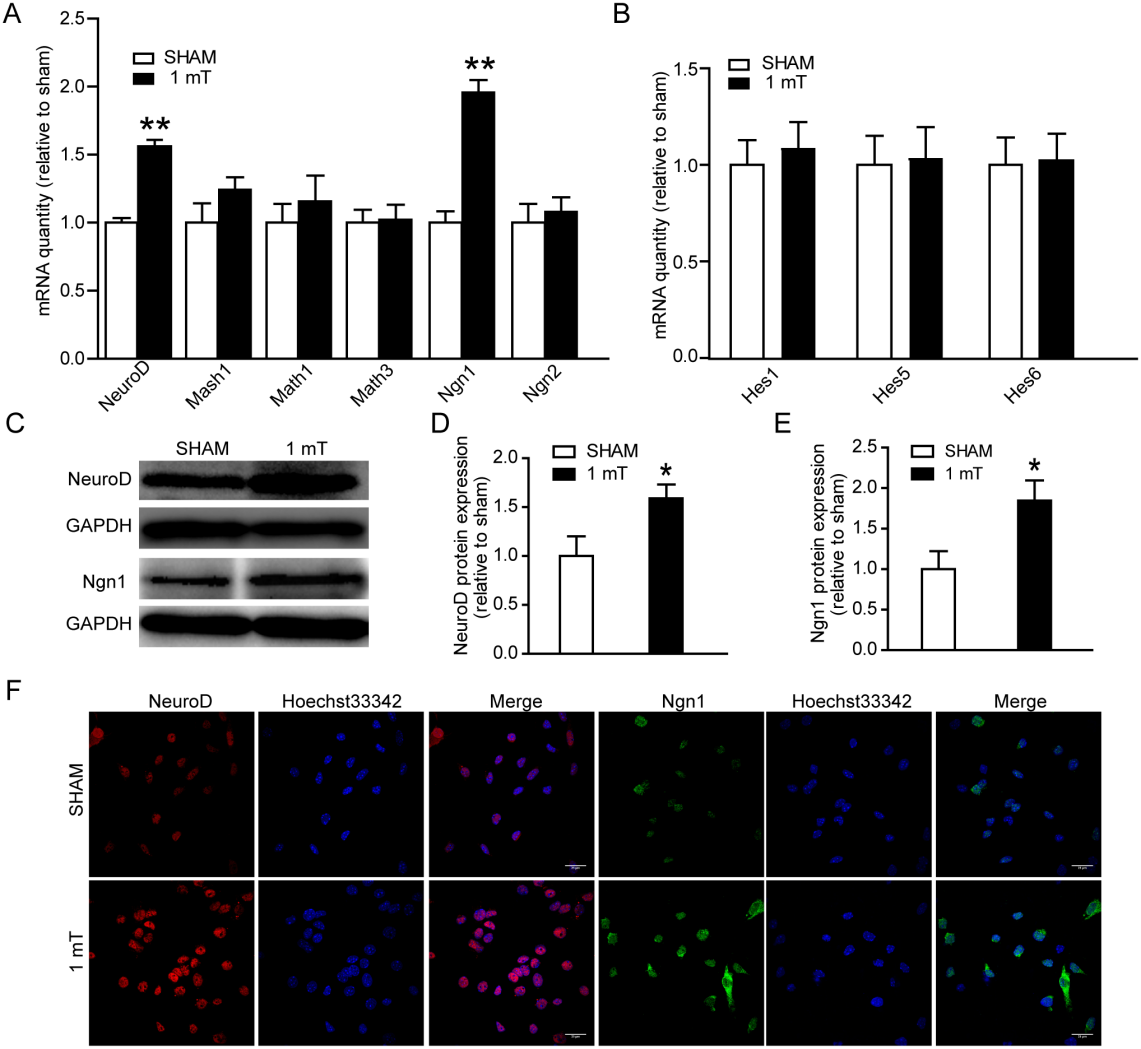
Effects of ELF-EMF exposure on bHLH genes expression. eNSCs were cultured in differentiation medium and exposed to ELF-EMF for 3 days. The mRNA expression of pro-neuronal genes (**A**) and Hes genes (**B**) was analyzed by real-time PCR. The mRNA expression of both NeuroD and Ngn1 was increased after ELF-EMF exposure. ** *p* < 0.01 vs. sham groups. (**C**) Representative western blotting bands of the NeuroD and Ngn1 protein expression. Full-length blots are presented in S4A and S4B Fig. (**D, E**) Statistical results of western blotting results. * *p* < 0.01 vs. sham groups. (**F**) Representative images of NeuroD and Ngn1 staining. For all experiments, the data are from five independent experiments and are presented as the mean ± SEM.

The bHLH transcription factors play key roles in controlling NSC differentiation and neurite outgrowth [34]. In our previous study, we found that part members of bHLH were changed after 1800 MHz radiofrequency EMF (RF-EMF) exposure [27]. Here, differentiating eNSCs were exposed to ELF-EMF for 3 days. Firstly, the mRNA expression of pro-neuronal genes (NeuroD, Mash1, Math1, Math3, Ngn1 and Ngn2) was measured using real-time PCR. We found that ELF-EMF exposure significantly increased the mRNA expression of NeuroD and Ngn1 (Fig 6A). Next, the mRNA expression of Hes genes (Hes1, Hes5 and Hes6), most of which are repressor bHLH genes during neuronal development, was measured. No change was observed (Fig 6B). Furthermore, the protein expression of NeuroD and Ngn1 was also measured using western blotting to determine whether ELF-EMFs also act at the translational level. Increased protein levels of NeuroD and Ngn1 were observed following ELF-EMF exposure (Fig 6C–6E). Additionally, the results of immunofluorescence staining showed that the expression of NeuroD and Ngn1 were significantly increased after ELF-EMF exposure (Fig 6F). These findings suggest that ELF-EMF exposure up-regulates pro-neuronal genes in differentiating eNSCs.

### Effects of ELF-EMF exposure on TRPC1 expression and function

**Fig 7:**
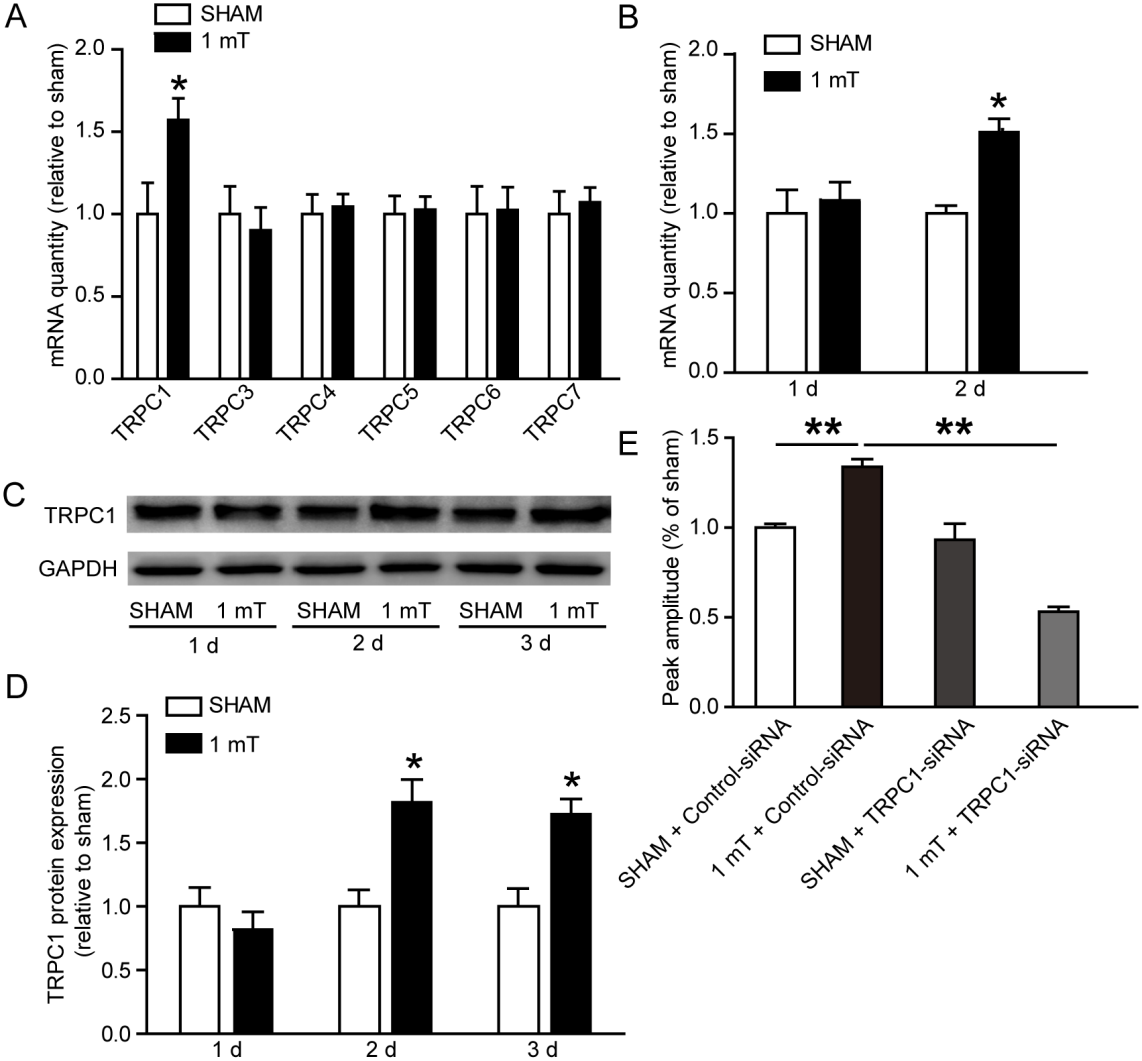
Effects of ELF-EMF exposure on TRPC1 expression and function. (**A**) mRNA expression of TRPC channels. Differentiating eNSCs were exposed to ELF-EMF for 3 days. * *p* < 0.05 vs. sham groups. (**B**) The mRNA expression of TRPC1 after ELF-EMF exposure for 1 day and 2 days. * *p* < 0.05 vs. sham groups. (**C, D**) Representative western blotting bands showing the TRPC1 protein expression and the statistical results. * *p* < 0.05 vs. sham groups. Full-length blots are presented in S4C Fig. (**E**) The changes in the intracellular Ca^2+^ concentration (indicated by F340/380 ratio) were measured with or without the TRPC1-siRNA treatment. The results were expressed as the peak amplitude relative to sham. ** *p* < 0.01. For all experiments, the data are from five independent experiments and are presented as the mean ± SEM.

Calcium channel plays an important role in the biological effect of EMF. It has been reported that TRPC channels expressed at high levels in the brain, are particularly important for embryonic neurodevelopment [35]. To determine whether ELF-EMF exposure affect TRPCs, we detected the mRNA expression of TRPC1 and TRPC3-7 (TRPC2 was not studied because it was a pseudo gene and was not detected in our previous study [36]) using real-time PCR in differentiating eNSCs after ELF-EMF exposure for 3 days. We only found a significant up-regulation of TRPC1 mRNA (Fig 7A). The TRPC1 mRNA expression was also detected after exposure to ELF-EMF for 1 and 2 days. We found that ELF-EMF exposure significantly increased the TRPC1 mRNA expression after the 2-day ELF-EMF exposure (Fig 7B). Furthermore, the Western blot results showed that the protein expression of TRPC1 was also significantly increased after the exposure to ELF-EMF for 2 and 3 days (Fig 7C and 7D).

Because EMF exposure could increased [Ca^2+^]_i_, we used Fura 2-AM to measure [Ca^2+^]_i_. The peak amplitude of [Ca^2+^]_i_ (indicated by the F340/380 ratio) was significantly increased after ELF-EMF exposure (Fig 7E). To determine whether TRPC1 participated in the alteration of intracellular calcium homeostasis, firstly, eNSCs were transfected with TRPC1-siRNA or control- siRNA. The results of both real-time PCR and western blot showed that the expression of TRPC1 was decreased robustly after TRPC1-siRNA transfection for 3 days, indicating a high efficiency of RNA silence (S1 Fig). Then, the cells were exposed to ELF-EMF for 3 days in differentiation medium. We found that the TRPC1-siRNA treatment lowered the increase in the peak amplitude of [Ca^2+^]_i_ induced by ELF-EMF exposure (Fig 7E). These results indicate that ELF-EMF exposure could up-regulate TRPC1 expression and increase the intracellular calcium levels.

### TRPC1-siRNA treatment prevents ELF-EMF-induced neuronal differentiation and neurite outgrowth

**Fig 8:**
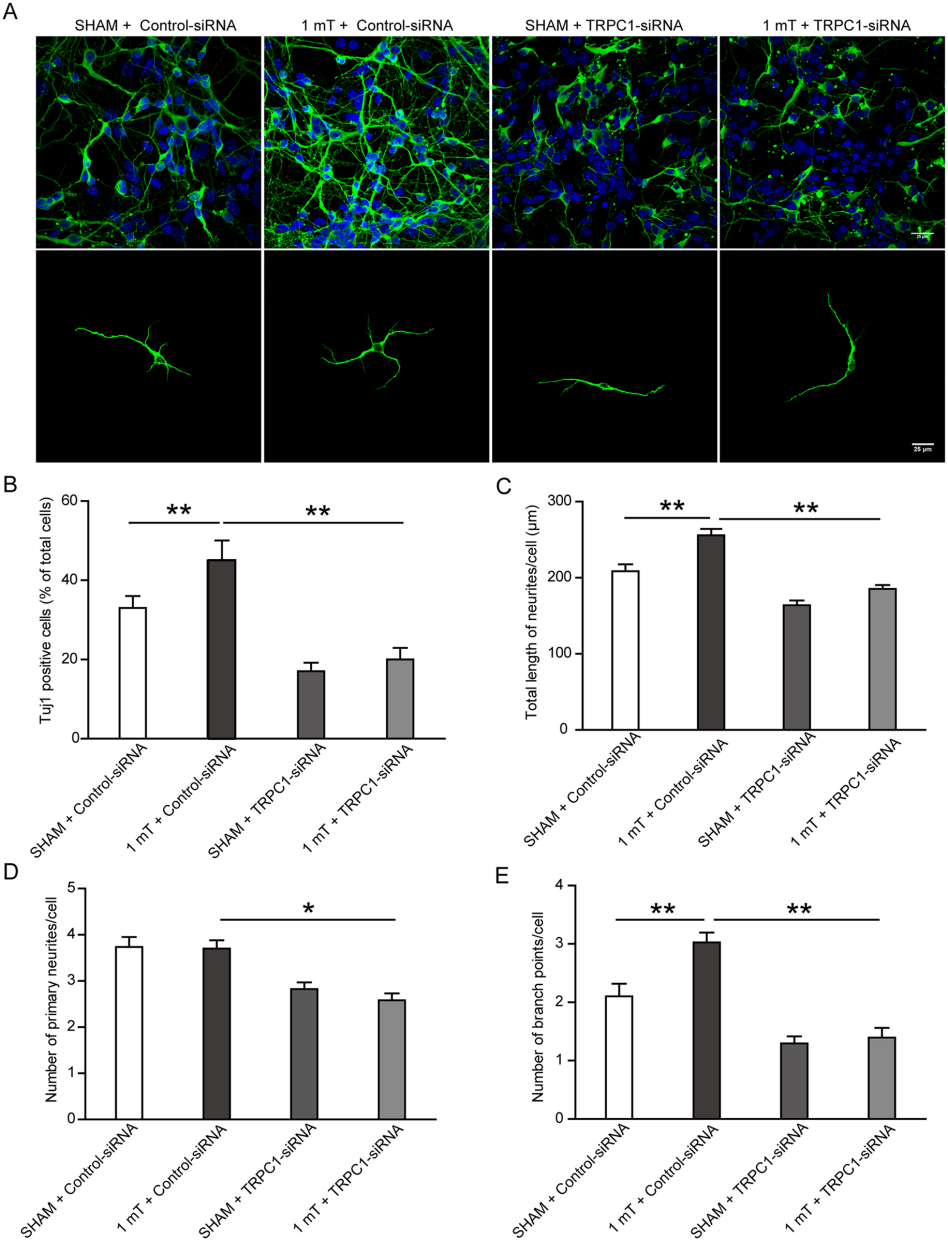
TRPC1-siRNA reduced ELF-EMF-induced neuronal differentiation and neurite outgrowth. eNSCs were transfected with TRPC1-siRNA or control-siRNA for 3 days to silence TRPC1 expression. Then the cells were cultured in differentiation medium and exposed to ELF-EMF for 3 days. The ratio and neurite outgrowth of eNSC-derived neurons were analyzed. (**A**) Representative images of Tuj1 staining. Scale bar: 25 μm. (**B**) Bar graphs showing the ratio of neurons differentiated from eNSCs. (**C-E**) Bar graphs showing the characteristics of the neurites. * *p* < 0.05 and ** *p* < 0.01. For all experiments, data are from five independent experiments and are presented as the mean ± SEM.

To further confirm that TRPC1 is responsible for the effects of ELF-EMF on the neuronal differentiation and neurite outgrowth of eNSCs, differentiating eNSCs, which were transfected with control-siRNA or TRPC1-siRNA, were exposed to ELF-EMF exposure for 3 days. We found that the ratio of neurons was significantly reduced in the sham-TRPC1-siRNA group as compared to the sham-control-siRNA group. Moreover, the TRPC1-siRNA treatment prevented the increase in the neuronal ratio induced by ELF-EMF exposure (Fig 8A and 8B). Similar alterations were also observed in the neurite outgrowth of eNSC-derived neurons (Fig 8A and 8C–8E). Additionally, we found that the TRPC1-siRNA treatment inhibited the mRNA expression of both NeuroD and Ngn1 (S2 Fig) and prevented their increase in response to ELF-EMF exposure in differentiating eNSCs. Collectively, these findings indicate that TRPC1 may be responsible for the effects of ELF-EMF exposure on the neuronal differentiation and neurite outgrowth of eNSCs.

## Discussion

eNSCs play a vital important role during embryonic brain development. eNSCs must undergo diverse processes, such as self-renewal, cell differentiation, cell apoptosis, and the development of neurites. Here, we found that eNSCs proliferation and maintenance were enhanced after ELF-EMF exposure *in vitro*. We also found that ELF-EMF exposure promoted neuronal differentiation and neurite outgrowth of eNSCs without inducing obvious cell apoptosis and TRPC1 participated in this process. These findings provide new evidence that ELF-EMF exposure can affect the behavior of eNSCs.

The ability of eNSCs proliferation and maintenance is of great important in maintaining stem cell pool for giving rise to diverse types of neurons and glia during brain development. Previous studies have reported that ELF-EMF (50 Hz, 1mT) exposure can increase cell proliferation in different cell models [37–39]. A facilitative effect on cell proliferation was also observed in NSCs from adult brain [11, 40]. However, the effects of ELF-EMF exposure on embryonic NSC proliferation were less unknown. In our study, we found that the proliferation of eNSCs was enhanced after ELF-EMF exposure with similar exposure parameters. Moreover, we observed that ELF-EMF exposure significantly facilitated the ability of eNSCs maintenance with increased numbers of primary and secondary neurospheres. Previous studies demonstrated that the up-regulation of vital genes (Sox2, Hes1 and Hes5) was associated with the enhancement of NSC proliferation and maintenance [41–45]. In our present study, we found that the mRNA expression of genes (Sox2, Hes1 and Hes5) was significantly increased following ELF-EMF exposure, indicating the enhanced proliferation of eNSCs induced by ELF-EMF. Therefore, our data suggest that ELF-EMF could enhance eNSCs proliferation and this facilitative effect may be a general biological effect of ELF-EMF (50 Hz, 1mT).

The cell differentiation of eNSCs is another pivotal process during embryonic neurogenesis. In our previous study, the ratio of neurons derived from eNSCs was not changed by the intermittent exposure to ELF-EMF (2 mT, 5 min on and 10 min off, 3 days) [9]. However, the present data showed that exposure to ELF-EMF (1 mT, 4 hours per day, 3 days) increased the ratio of eNSC-derived neurons. This promoting action of ELF-EMF exposure on neuronal differentiation was also reported in postnatal and adult NSCs at the same magnetic strength despite the difference in exposure time [10, 11]. The magnetic field strength and the exposure duration may account for the differences in neuron differentiation. However, we did not observe significant alterations in the ratio of eNSCs-derived astrocytes in our present study. In addition, we found no significant cell apoptosis in differentiating eNSCs. Combining the acceleration of ELF-EMF on eNSCs proliferation, these results indicate that ELF-EMF could facilitate neuronal differentiation of eNSCs. Further studies are still needed to verify this effect *in vivo* and clarify the possible mechanism.

Neurite outgrowth, another important process in brain development, is related to nerve fiber projection, synapse formation, and neuron maturation. Previous studies reported that ELF-EMF exposure could alter the neurite outgrowth (e.g., the percentage of neurite-bearing cells, the average length of neuritis and the direction of neurite outgrowth, et al) of the PC12 cell and dorsal root ganglia in a frequency- and density-dependent manner [46, 47]. Moreover, we reported that exposure to 1800 MHz RF-EMF impaired the neurite outgrowth of eNSC-derived neurons [27]. On the basis of these findings, we measured the neurite outgrowth of eNSC-derived neurons after ELF-EMF exposure and found the increases in both the total length of neurites per cell and the number of branch points per cell, although the number of primary neurites was not changed. This is the first to report the effects of ELF-EMF exposure on the neurite outgrowth of eNSCs-derived neurons. These findings suggest that the neurite outgrowth of eNSCs may be susceptible to the effects of EMFs.

In the developing brain, pro-neuronal bHLH factors, such as Ngn1, Ngn2, Mash1, NeuroD, Math1, and Math3, are key regulators of neurogenesis and coordinate the neuronal fate specialization and neurite outgrowth of eNSCs [34, 48]. In our previous studies, we found that 1800 MHz RF-EMF exposure impaired the neurite outgrowth of eNSCs by disrupting bHLH expression. The pro-neuronal genes Ngn1 and NeuroD were down-regulated [27]. Thus, we hypothesize that changes in pro-neuronal genes may also contribute to the promotion of neuronal differentiation and neurite outgrowth observed in eNSCs exposed to ELF-EMFs. Interestingly, our data showed a significant up-regulation of these two genes in response to ELF-EMF exposure. Our results are also supported by those of Cuccurazzu and Leone, who reported that exposing adult NSCs to ELF-EMF resulted in up-regulation in Ngn1 and NeuroD [11, 49]. These results further indicate that bHLH factors are involved in the ELF-EMF-induced neuronal differentiation and neurite outgrowth of eNSCs.

The above findings prompt the following question: how does ELF-EMF exposure up-regulate pro-neuronal genes and cause the neuronal differentiation and neurite outgrowth of eNSCs? EMF-mediated extracellular Ca^2+^ influx is one of the main mechanisms of EMF biological effects. Previous studies reported that ELF-EMF exposure increased the expression and activity of a Ca_v_1(L-type) channel (belonging to VOCCs) and promoted the neuronal differentiation of postnatal and adult NSCs [10, 11]. However, eNSCs express few classical VOCCs. Therefore, non-VOCCs may also play critical roles in ELF-EMF exposure-induced neuronal differentiation and neurite outgrowth of eNSCs. The mammalian TRPC family, a class of non-VOCCs, participates in the proliferation and neuronal differentiation of NSCs and different members of TRPC family regulate neurite extension [36, 50]. In PC12 cells, TRPC1 promoted neurite outgrowth, whereas TRPC5 reduced neurite outgrowth [21]. TRPC5 played a key role in the switch between the proliferation and neuronal differentiation of neural progenitor cells [20]. A TRPC1/TRPC3-mediated increase in store-operated calcium entry (SOCE) was observed in the differentiation of H19-7 hippocampal neuronal cells [51]. TRPC6 promoted dendritic growth via the CaMKIV-CREB pathway [52]. In our study, we found that Tuj1^+^ neuron derived from eNSCs expressed TRPC1 (S3 Fig), indicating that TRPC1 play a role in neuronal differentiation. ELF-EMF exposure significantly up-regulated TRPC1 expression and increased [Ca^2+^]_i_. The combination of ELF-EMF exposure with TRPC1-siRNA treatment decreased [Ca^2+^]_i_ compared with that of ELF-EMF exposure alone. Due to the limitations of our experimental conditions, we did not measure [Ca^2+^]_i_ during ELF-EMF exposure. However, our results suggest that TRPC1 contributes to ELF-EMF induced [Ca^2+^]_i_ increase.

Changes in the intracellular Ca^2+^ concentration mediated by ion channels and receptors regulate the expression of several bHLH transcription factors [32, 33, 48]. In our study, TRPC1-siRNA treatment prevented the mRNA up-regulation of proneural genes (NeuroD and Ngn1) and prevented the enhancement of neuronal differentiation and neurite outgrowth of eNSCs in response to ELF-EMF exposure. These results further support the role of the TRPC1 channel and the corresponding calcium signal in the effects of ELF-EMF exposure on eNSCs. The detailed molecular mechanism of how TRPC1-mediated calcium signaling changes pro-neuronal gene expression and the final neuronal differentiation and neurite outgrowth of eNSCs must still need investigation.

Here, combining with previous studies, we would like to discuss the potential mechanisms underlying the effects of ELF-EMF exposure on NSCs. When NSCs are exposed to ELF-EMF, the increase of the intracellular Ca^2+^ levels may play as an initial factor in subsequent cell behaviors [13, 14, 53–55]. The intracellular Ca^2+^ may be from the extracellular Ca^2+^ influx through the receptors [12] and the calcium channels [10, 11, 38, 56]. The effects of ELF-EMF on NSCs (proliferation and differentiation) were linked to enhanced expression and function of voltage-gated L-type calcium channels (Cav1) [10, 38]. This indicated that ELF-EMF might also affect both the expression and function of TRPC1 channels. Then the increase in the intracellular Ca^2+^ levels will enhance pro-neuronal bHLH gene expression (i.e., NeuroD and Ngn1) by mechanisms involving Ca^2+^-dependent phosphorylation of cAMP response element binding protein (CREB) [11], CREB/CREB-binding protein (CBP) recruitment on the pro-neuronal gene promoters and histone acetylation on the regulatory sequence of these genes [49, 57]. Indeed, the mechanisms of ELF-EMF exposure on NSCs are beyond the above mentioned and still needed further researches.

In conclusion, we found that TRPC1 is involved in ELF-EMF-induced neuronal differentiation and neurite outgrowth of eNSCs *in vitro*. Although the promotion of neurogenesis in adult mice exposed to ELF-EMF appears to be beneficial, embryonic brain development is different from adulthood. A subtle alteration during this period may lead to severe impairment in adulthood. Therefore, it is not clear that the promotion of ELF-EMF exposure on neuronal differentiation and neurite outgrowth of eNSCs is beneficial or harmful. Further studies are required to evaluate the potential effects of ELF-EMF exposure on embryonic brain development.

## Supporting Information

S1 Fig The knockdown efficiency of TRPC1-siRNA. The knockdown efficiency of TRPC1-siRNA. (**A**) The mRNA expression of TRPC1 after TRPC1-siRNA transfected for 3 days. n = 5, ** p < 0.01. (**B, C**) The protein expression of TRPC1. eNSCs were transfected with TRPC1-siRNA for 3 days. The protein expression of TRPC1 was detected at 1 day and 3 days after differentiation. The left panel and the right panel are from the same membrane. n = 5, ** *p* < 0.01. For all experiments, data are presented as the mean ± SEM.
(TIF)

S2 Fig TRPC1-siRNA eliminates the effects of ELF-EMF on NeuroD and Ngn1 expression. TRPC1-siRNA eliminates the effects of ELF-EMF on NeuroD and Ngn1 expression. eNSCs were first transfected with TRPC1-siRNA or control-siRNA for 3 days to silence TRPC1 expression. Then the cells were cultured in differentiation medium and exposed to ELF-EMF for 3 days. (**A**) The mRNA expression of the NeuroD gene. (**B**) The mRNA expression of the Ngn1 gene. ** *p* < 0.01. For all experiments, the data are from five independent experiments and are presented as the mean ± SEM.
(TIF)

S3 Fig Tuj1 positive neuron expressed TRPC1. Tuj1 positive neuron expressed TRPC1. eNSCs were cultured in differentiation medium for 3 days. The cells were then fixed for Tuj1 and TRPC1 staining. Scale bar: 25 μm.
(TIF)

S4 Fig Full length image of the blots of NeuroD, Ngn1, and TRPC1. Full length image of the blots of NeuroD, Ngn1, and TRPC1. (**A**) Full length image of the blots of NeuroD (The NeuroD antibody (sc-398891) is purchased from Santa Cruz Corp. The real position of NeuroD in the membrane showed small bias compared to its molecular weight (50 kDa). However, the position is consistent with the representative western blotting band in antibody instruction). (**B**) Full length image of the blots of Ngn1 (26 kDa). (**C**) Full length image of the blots of TRPC1 (92 kDa). The left panel and the right panel in A-C are from the same membrane.
(TIF)
