# Supplementary figures and images for: Extremely Low-Frequency Electromagnetic Fields Promote In Vitro Neuronal Differentiation and Neurite Outgrowth of Embryonic Neural Stem Cells via Up-Regulating TRPC1
Source: PLoS One. 2016 Mar 7;11(3):e0150923. doi: 10.1371/journal.pone.0150923 (PMC4780708; doi:10.1371/journal.pone.0150923)

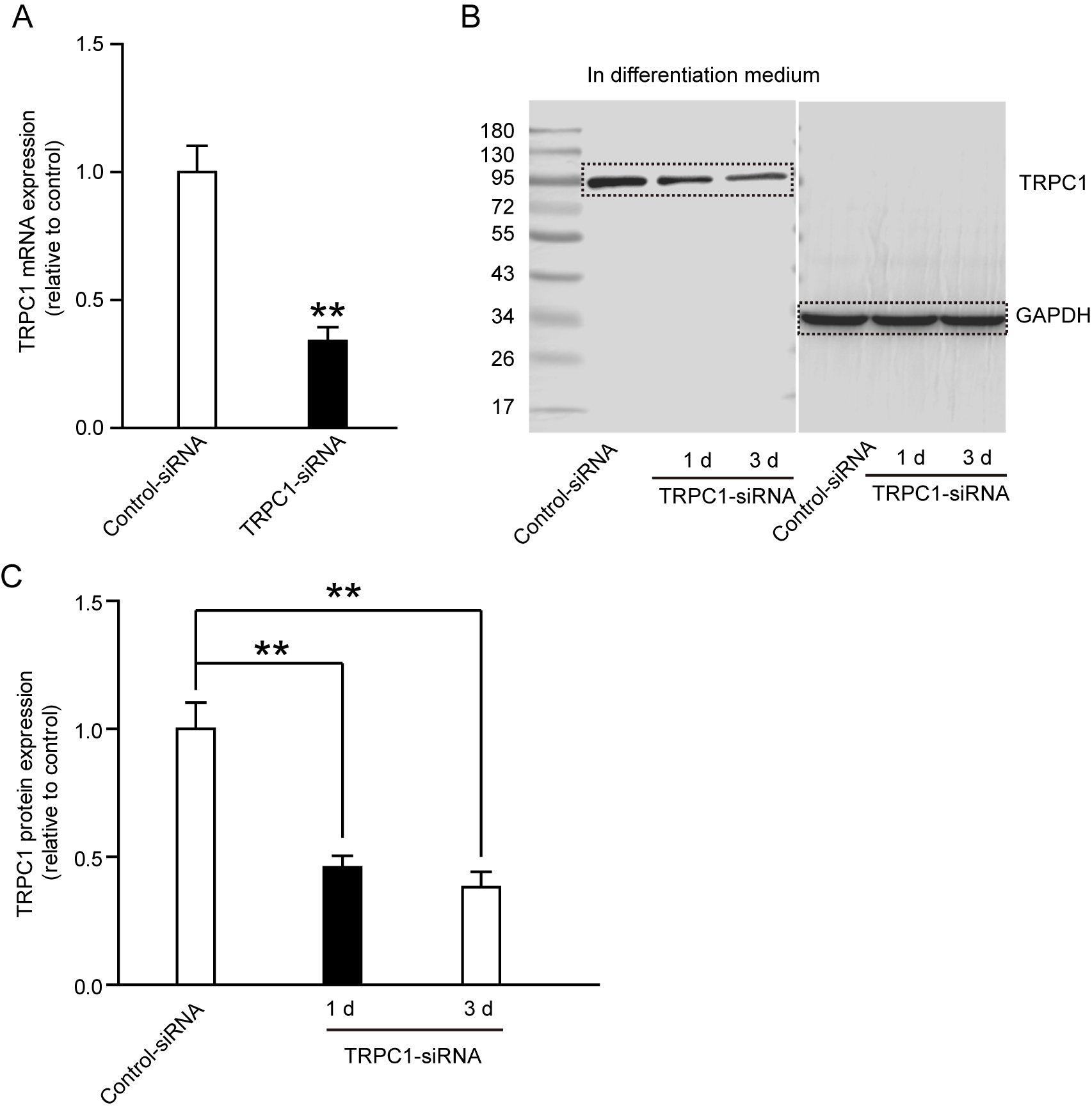

Supplement: S1 Fig — The knockdown efficiency of TRPC1-siRNA. (A) The mRNA expression of TRPC1 after TRPC1-siRNA transfected for 3 days. n = 5, ** p < 0.01. (B, C) The protein expression of TRPC1. eNSCs were transfected with TRPC1-siRNA for 3 days. The protein expression of TRPC1 was detected at 1 day and 3 days after differentiation. The left panel and the right panel are from the same membrane. n = 5, ** p < 0.01. For all experiments, data are presented as the mean ± SEM. (TIF) [file pone.0150923.s001.tif]

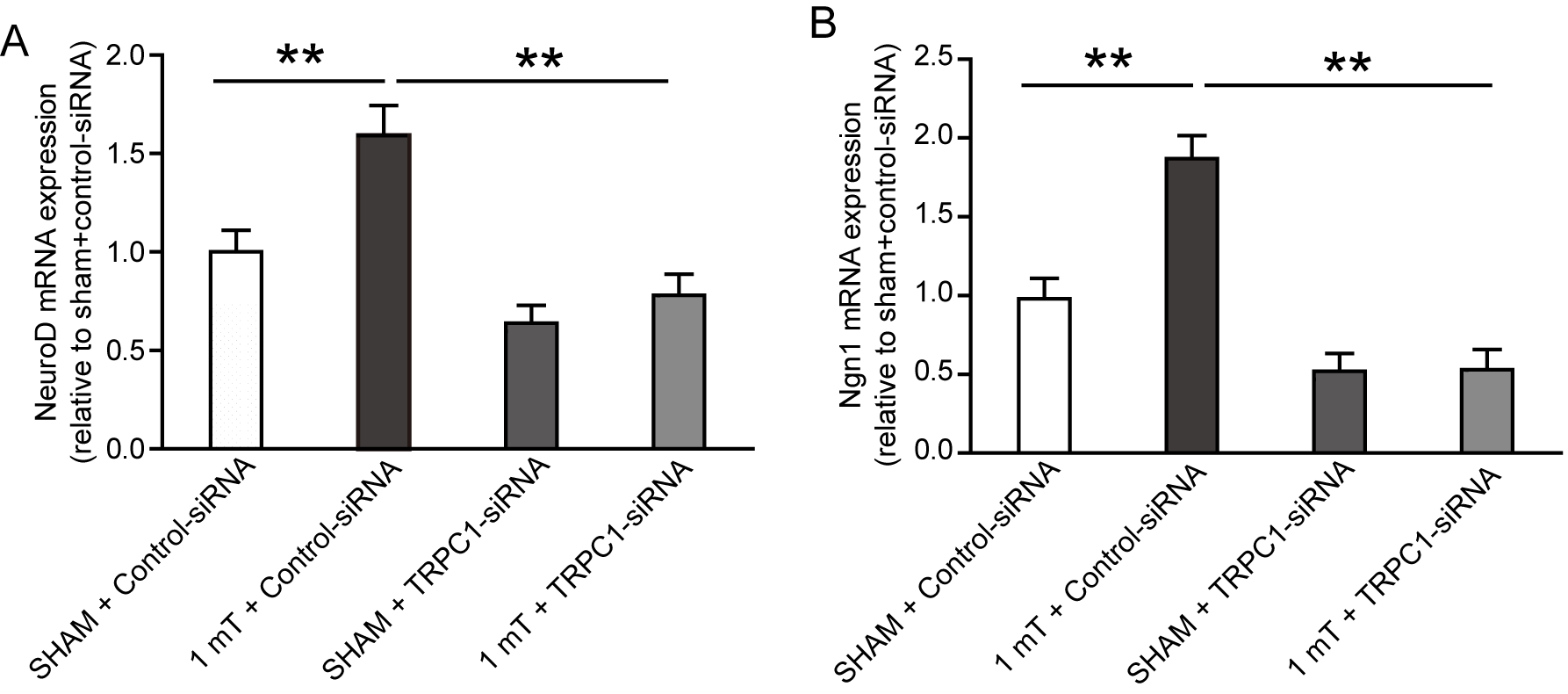

Supplement: S2 Fig — TRPC1-siRNA eliminates the effects of ELF-EMF on NeuroD and Ngn1 expression. eNSCs were first transfected with TRPC1-siRNA or control-siRNA for 3 days to silence TRPC1 expression. Then the cells were cultured in differentiation medium and exposed to ELF-EMF for 3 days. (A) The mRNA expression of the NeuroD gene. (B) The mRNA expression of the Ngn1 gene. ** p < 0.01. For all experiments, the data are from five independent experiments and are presented as the mean ± SEM. (TIF) [file pone.0150923.s002.tif]

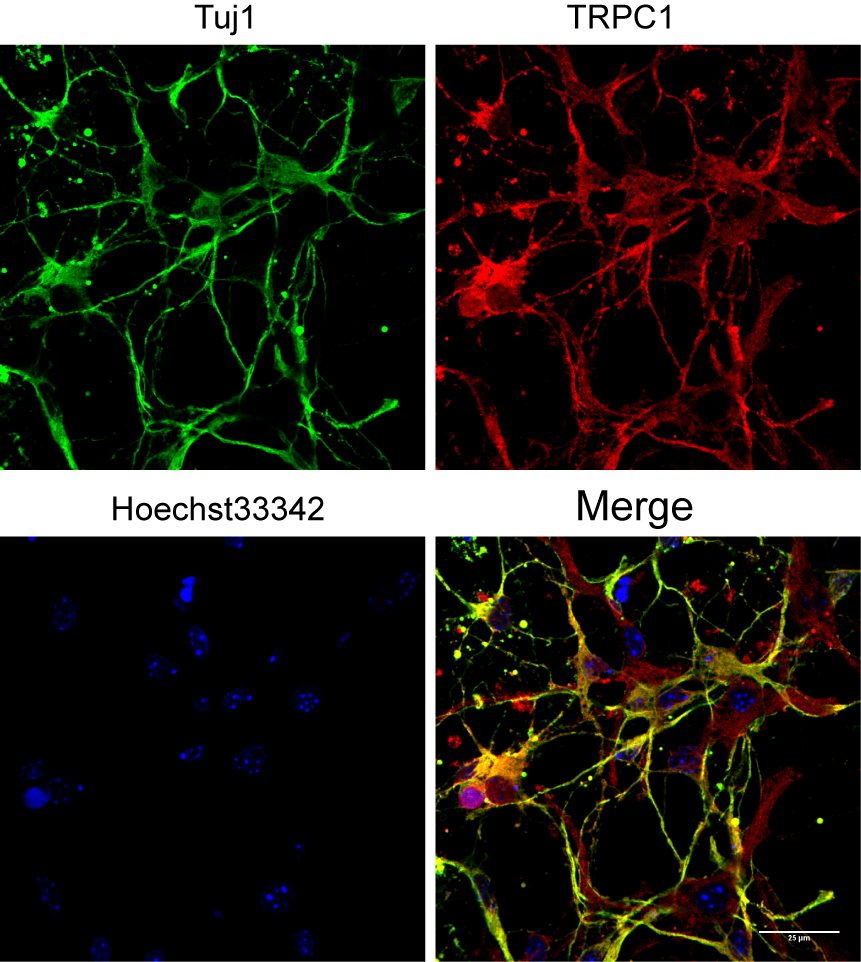

Supplement: S3 Fig — Tuj1 positive neuron expressed TRPC1. eNSCs were cultured in differentiation medium for 3 days. The cells were then fixed for Tuj1 and TRPC1 staining. Scale bar: 25 μm. (TIF) [file pone.0150923.s003.tif]

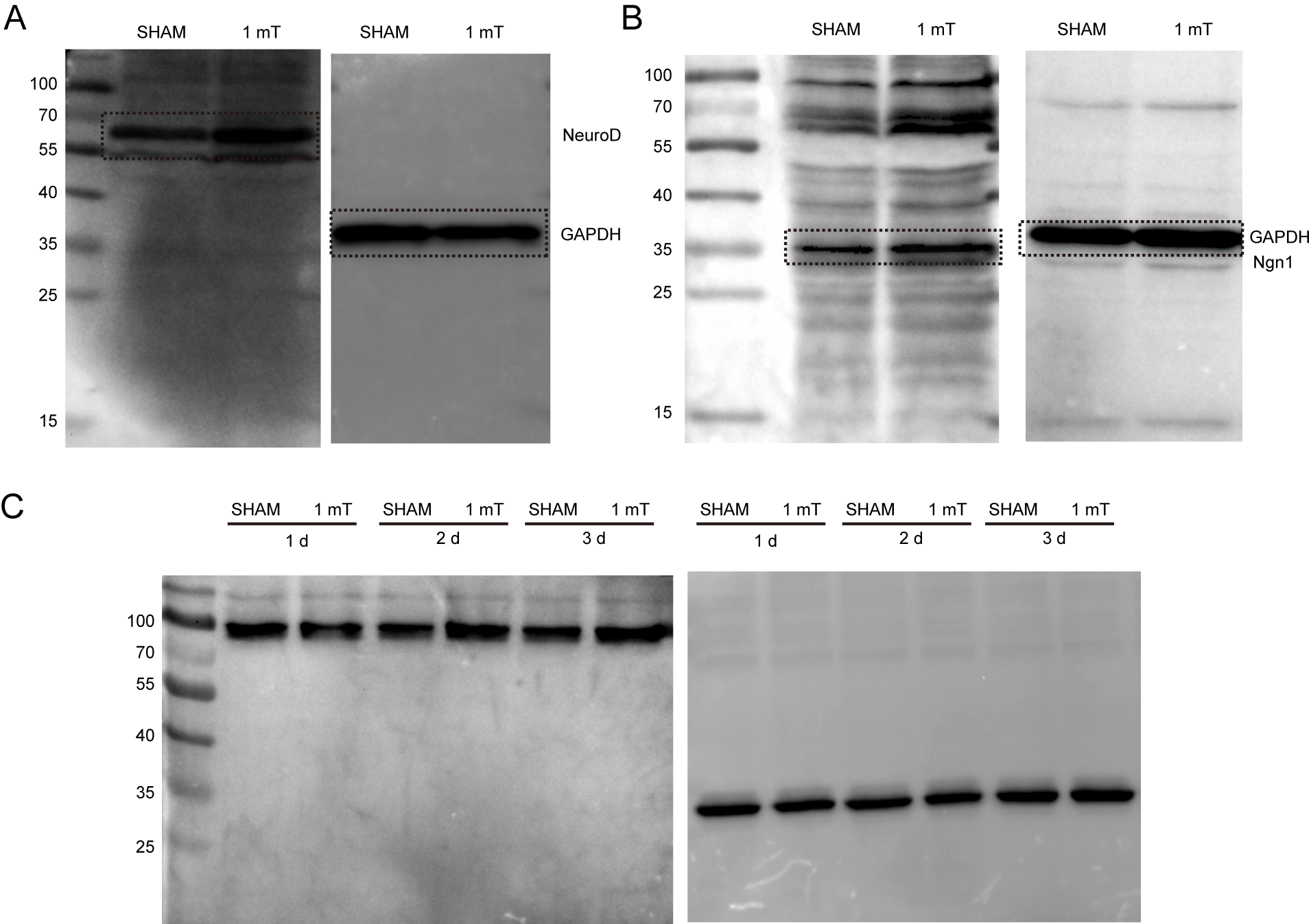

Supplement: S4 Fig — Full length image of the blots of NeuroD, Ngn1, and TRPC1. (A) Full length image of the blots of NeuroD (The NeuroD antibody (sc-398891) is purchased from Santa Cruz Corp. The real position of NeuroD in the membrane showed small bias compared to its molecular weight (50 kDa). However, the position is consistent with the representative western blotting band in antibody instruction). (B) Full length image of the blots of Ngn1 (26 kDa). (C) Full length image of the blots of TRPC1 (92 kDa). The left panel and the right panel in A-C are from the same membrane. (TIF) [file pone.0150923.s004.tif]
